# The value of vestibular graviceptive pathway evaluation in the diagnosis of unilateral peripheral vestibular dysfunction

**DOI:** 10.1002/brb3.3055

**Published:** 2023-05-15

**Authors:** Tong‐tong Zhao, Meng‐lu Zhang, Yu‐fei Feng, Qian‐qian Wang, Ning Song, Xu Yang, Xiao‐hong Ba

**Affiliations:** ^1^ Department of Neurology The First Affiliated Hospital of Jinzhou Medical University Jinzhou China; ^2^ Department of Neurology Aerospace Center Hospital, Peking University Aerospace School of Clinical Medicine Beijing China

**Keywords:** ocular tilt reaction, subjective visual horizontal, subjective visual vertical, unilateral peripheral vestibular dysfunction, vestibular graviceptive pathway

## Abstract

**Background:**

Evaluation of vestibular graviceptive pathway (VGP) in patients with unilateral peripheral vestibular dysfunction (UPVD) has received increasing attention from researchers. The study aimed to investigate the value of VGP evaluation in the diagnosis of UPVD.

**Methods:**

Ninety‐five UPVD patients were divided into attack and remission phase groups. VGP evaluation‐related indicators, including subjective visual vertical (SVV), subjective visual horizontal (SVH), head tilt, ocular torsion (OT), and skew deviation (SD), were measured, and their correlations with cochleovestibular function test results were analyzed. The possible etiologies of contralesional VGP (c‐VGP) were analyzed.

**Results:**

Positive rates of SVV, SVH, OT, and SD were significantly higher, and the degrees of SVV, SVH, and OT were significantly greater in the attack phase group than the remission phase group. The sides with abnormal VGP evaluation results were correlated with the sides with hearing loss, abnormal caloric, and video head impulse test (vHIT) results. A total of 14 patients showed c‐VGP, and possible etiologies included contralateral benign paroxysmal positional vertigo (*n* = 4), bilateral hearing loss (*n* = 8), bilateral vHIT gain reduction (*n* = 1), autoimmune diseases (*n* = 6), vascular risk factors (*n* = 6), lacunar infarction (*n* = 3), and endolymphatic hydrops (*n* = 3).

**Conclusions:**

Alterations in SVV, SVH, OT, and SD were noted in UPVD patients in different phases, which are presumed to be related to dynamic vestibular compensation; correlations between VGP evaluation results and cochleovestibular function test results indicate that VGP evaluation may be helpful for the diagnosis of the side affected in UPVD; the presence of c‐VGP may be related to bilateral labyrinth lesions or endolymphatic hydrops on the affected side; and the involvement of autoimmune mechanisms also deserves attention.

## Graphical abstract

(1) Alterations in SVV, SVH, OT, and SD were noted in patients with UPVD in acute attack and remission phases, which are presumed to be related to dynamic vestibular compensation. (2) There were significant correlations between VGP evaluation results and semicircular canal function test, audiological test results, indicating that VGP evaluation may be helpful for localization diagnosis of side involvement in patients with UPVD. ( 3) The presence of c‐VGP in UPVD patients may be mostly related to bilateral labyrinth lesions or endolymphatic hydrops on the affected side, and the involvement of autoimmune mechanisms also deserves attention.

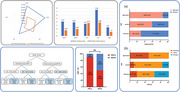

## 1 INTRODUCTION

Vertigo is a disorder of body's sense of balance and orientation in space, and an illusion of movement of the person or of the external world. Clinically, the etiologies of vertigo/vestibular disorders are complex, which involves multidisciplinary knowledge. In a population‐based questionnaire study, approximately 20% to 30% of the population experienced dizziness/vertigo symptoms. A national survey of Germans showed that the lifetime prevalence of vertigo was 7.4% in adults aged 18–79 years, which was more common in women, with a female to male prevalence ratio of 2.7:1, and the prevalence of vertigo increased obviously with age (Strupp et al., 2020). In the United States, there are nearly 10 million visits to emergency departments each year for complaints of dizziness or vertigo, accounting for about 25% of all emergency department visits (Saber Tehrani et al., 2013).

In fact, damage to the unilateral peripheral vestibular pathway (including vestibular hair cells and vestibular nerve) caused by any physical or chemical factors can lead to asymmetric damage to the bilateral vestibular system, resulting in corresponding symptoms of imbalance, such as dizziness/vertigo, spontaneous nystagmus, severe nausea, and vomiting, this is called unilateral peripheral vestibular dysfunction (UPVD). A German multicenter study of 34,860 patients with dizziness/vertigo showed a high incidence of UPVD (approximately 9.1%) (Strupp et al., 2020). Patients with UPVD generally experience a rapid onset, most of these patients visit the hospital due to persistent dizziness/vertigo, and UPVD is often accompanied by clinical symptoms and signs such as spontaneous nystagmus, instability, nausea, and vomiting. For years, clinicians have relied mainly on semicircular canal function tests, such as caloric test, rotatory chair tests, and video head impulse test (vHIT), to determine the affected side of UPVD. A study has shown that up to 40% of patients with dizziness/vertigo showed unilateral canal paresis (CP) during caloric test (Goebel & Paige, 1989). In recent years, with the rapid development of the anatomic basis of the vestibular system, clinical theories, and related research tools, the evaluation of vestibular graviceptive pathway (VGP) based on the impairment of utricular function and pathways in patients with UPVD has received more and more attention from researchers.

In clinical practice, VGP evaluations mainly include subjective visual vertical (SVV), subjective visual horizontal (SVH), head tilt (HT), ocular torsion (OT), and skew deviation (SD). Studies have found that 80%–94% of patients with UPVD showed SVV/SVH tilts toward the affected side, and about 10% of patients showed SVV/SVH tilts toward the unaffected side (Faralli et al., 2014). Hirvonen et al. (2011) and Strupp (2008) showed that 60% of patients with acute vestibular neuritis (VN) had HT to the affected side, and the inaccurate perception of gravity may be further exacerbated by head movement on the affected side. Faralli et al. (2021) showed that in most of the patients with superior VN, the SVV tilt values returned to normal range within 3–6 months, whereas OT remained abnormal after 1 year or even longer, suggesting that OT may be a good indicator of the entity of the residual peripheral otolithic lesion. In fact, it is still controversial whether OT or SVV recovers faster (Müller et al., 2016). Gufoni et al. (2020) found that the measurement of SD can determine when mechanical damage to the utricule may have occurred in patients with canalolithiasis.

In clinical practice, VGP evaluation is of great clinical value in the diagnosis of the affected side in patients with UPVD, but its clinical manifestations and value in the attack and remission phases of UPVD and the etiology of contraversive SVV/SVH deserve further investigations. Given this background, the present study investigated the changes in five VGP evaluation indicators (SVV, SVH, HT, SD, and OT) in 95 patients with UPVD (including 46 patients in the attack phase, and 49 patients in the remission phase) analyzed their correlations with related vestibular symptoms and vestibular function test results, so as to provide clinical evidence for the use of VGP evaluation in the diagnosis of UPVD.

## 2 MATERIAL AND METHODS

### 2.1 Patients

**FIGURE 1:**
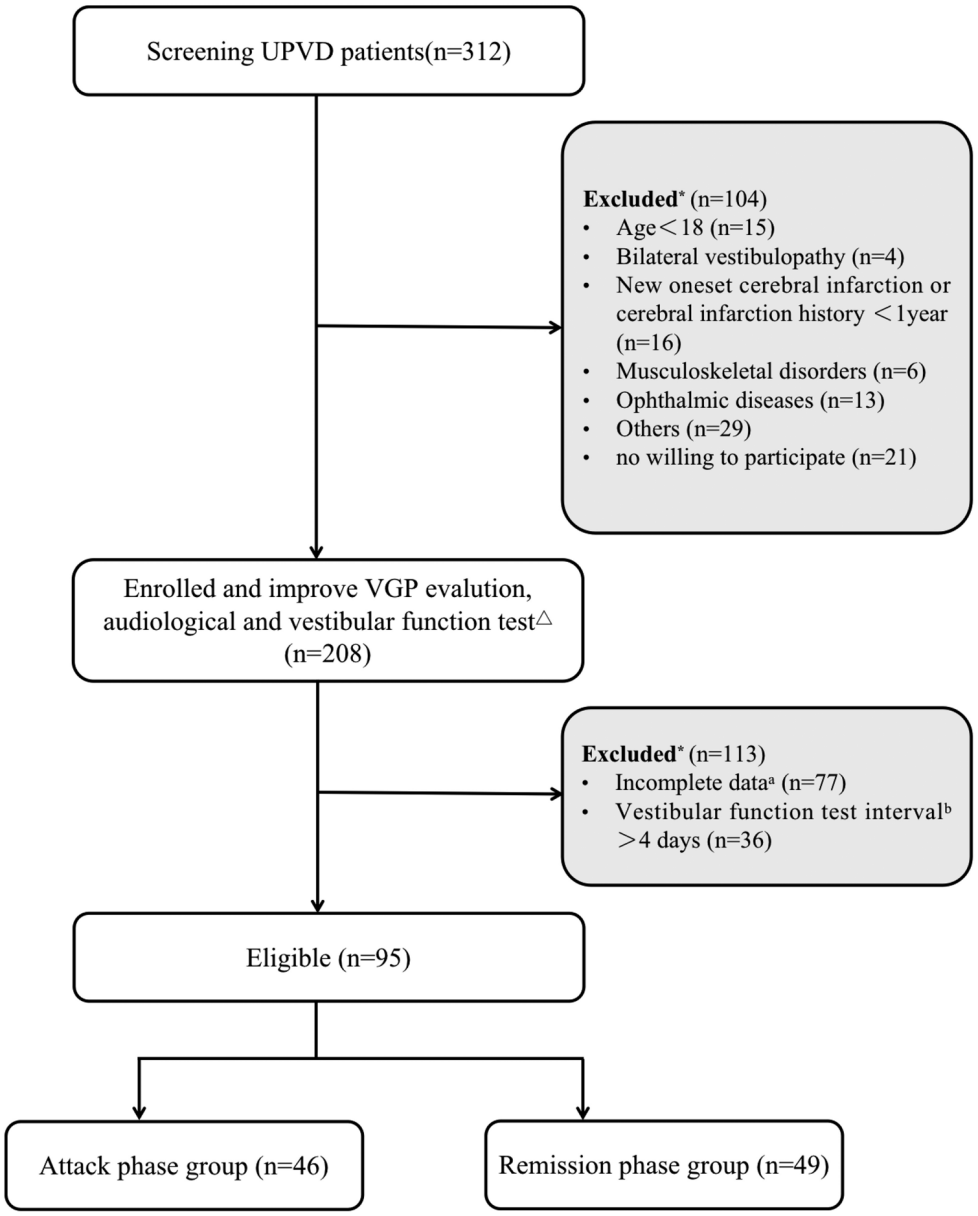
Flowcharts for study screening and enrollment process. ^*^: Patients had one or several reasons for exclusion. ^△^: The vestibular function tests in this study included caloric test, vestibular graviceptive pathway (VGP) evaluation, video head impulse test (vHIT), and ocular vestibular evoked myogenic (oVEMP). ^a^: Patients who dropped out and failed to complete all 5 VGP evaluation were considered incomplete data. ^b^: Patients completed vestibular function tests interval >4 days. If the patient's caloric test and VGP evaluation interval >4 days, the patient is excluded; if the patient's caloric test and VGP evaluation interval ≤4 days, the caloric test and vHIT/oVEMP >4 days, only the caloric test and VGP data of the patient were used, and the vHIT/oVEMP data were excluded.

A prospective, cross‐sectional study design was implemented in a tertiary hospital with an advanced vertigo center in Beijing from March 04, 2021 to May 14, 2022. The population selection targets those patients with UPVD. In total, 312 patients were screened, of which 208 met the inclusion criteria and were enrolled. The inclusion criteria included (1) unilateral CP of >25% on caloric test; (2) age ≥18 years old. Patients were excluded when they had the following conditions: (1) Bilateral vestibulopathy met the diagnostic criteria of the Barany Society; (2) new onset cerebral infarction or cerebral infarction history <1 year; (3) patients who had ophthalmoplegia or lesions of the oculomotor nucleus, scoliosis, or pelvic tilt that may affect VGP evaluation; (4) patients who did not sign the informed consent; (5) patients who dropped out and failed to complete all 5 VGP evaluation; (6) vestibular function tests (caloric test, VGP evaluation, vHIT, ocular vestibular evoked myogenic potential [oVEMP]) interval >4 days. This study has no missing data. Figure 1 shows the inclusion flowchart for this study. All patients completed medical history, physical examination, video‐nystagmography (VNG) (to evaluation vestibular nystagmus and eye movements), calorie test, VGP evaluation, and other vestibular function tests within 4 days of attending the clinic, with a mean interval of 2 days. The time required for VNG is 20–30 min, calorie test is 10–20 min, all 5 VGP tests are 20–30 min, vHIT is 10–20 min, and oVEMP is 15–25 min.

According to the presence or absence of spontaneous nystagmus and the onset duration, patients were divided into two groups: attack and remission phase groups. The attack phase of UPVD was defined as the presence of spontaneous nystagmus or onset duration of ≤7 days. The remission phase of UPVD was defined as the absence of spontaneous nystagmus and onset duration of >7 days. Finally, 46 patients of attack UPVD patients (21 men, mean age 52.32 years old, range 21–75 years old) and 49 patients of remission UPVD (19 men, mean age 46.84 years old, range 24–70 years old) were enrolled.

This study was approved by the ethics committee of Aerospace Center Hospital (Peking University Aerospace School of Clinical Medicine). Written informed consent was obtained from all patients.

### 2.2 Caloric test

Spontaneous nystagmus, eye movements, and caloric‐induced nystagmus were recorded with the VNG (Interacoustics, Middelfart, Denmark). During the test, patients were positioned in the supine position in the semi‐dark room, with their head tilted up about 30° and with goggles placed over their eyes. The right and left external auditory canals were irrigated with once hot air at 50°C and once cold air at 24°C, respectively, for 1 min. The caloric irrigation was performed in the following order: right ear warm (50°C, RW), left ear warm (50°C, LW), right ear cold (24°C, RC), left ear cold (24°C, LC), and the between‐irrigation interval was 5 min. Each irrigation was only performed until the nystagmus had completely disappeared. The sides of strong and weak caloric responses were determined by the maximum slow phase velocity (SPV) values of the total responses from the left and right ears, that is, absolute SPV values of RC + RW responses versus the absolute SPV values of LC+LW responses. CP value was calculated according to the following formula: CP = (RW + RC) − (LW + LC)/(RW + RC + LW + LC) × 100. Unilateral CP > 25% was defined as unilateral horizontal semicircular canal dysfunction (Zingler et al., 2007).

### 2.3 vHIT

vHIT (Interacoustics, Middelfart, Denmark) was used to evaluate the function of the three pairs of semicircular canals. The instrument comprises an inertial measurement unit to measure head movements and an infrared camera to record eye movements. In a brightly lit room, patients were in the sitting position and wore an eye mask. There were instructed to fix their eyes on a target at eye level and at a distance of 1.5 m. The examiner stood behind the patient while holding the patient's head between both hands. A total of 20 head impulses were delivered in each canal plane in a brief, rapid, passive manner; the angular velocity was 150–250°/s for the horizontal canal impulses, 100–200°/s for the vertical canal impulses, and the amplitude was 10–20°. The vHIT software was used to record the average slow phase vestibulo‐ocular reflex (VOR) gain values (the ratio of eye velocity to head velocity at 60 s) and saccades (McGarvie et al., 2015).

### 2.4 VGP evaluation

#### 2.4.1 SVV and SVH

SVV was tested using an SVV measuring instrument (ZT‐SVV‐I, Shanghai ZEHNIT Medical Technology Co., Ltd., Shanghai, China). Subjects were seated upright with their heads in an erect neutral position and wore a contour mask that restricted the visual field to eliminate visual references. A yellow luminous bar with a length of 60 cm was projected on a black background of a visual field in front of patients (distance: 2 m), and an initial position of the bar was set at ±25°. SVV and SVH were defined as 0° based on the gravitational vertical and horizontal lines, respectively, and the two lines were used as vertical and horizontal coordinates. The circular field of view was divided into four quadrants, the tilts of the vertical and horizontal axes to the right/upward in quadrants 1 and 2 were designated recorded as positive, and tilt to the left/downward was designated as negative. Patients were asked to adjust the angle of the bar with a control joystick with an accuracy of ±0.1°. Before the tests were started, patients were allowed to practice twice to familiarize themselves with the tests. In order to eliminate the influence of visual memory, the values were recorded from the third time of test. SVV and SVH tests were repeated seven times, respectively, and the average value was recorded.

#### 2.4.2 HT

The patient was instructed to keep the body and head straight as far as possible before the examination, whereas the tester observed the posture of the patient. If the height of the patient's shoulders was not consistent, the patient was verbally prompted to adjust the height of the shoulders to keep the shoulders at the same level. After the adjustment, the tester measured the angle between the sagittal axis of the patient's head and gravity with the iPhone protractor, which was the HT degree (Brandt & Strupp, 2005). HT was measured separately by two experienced neurologists, and the average of the two measurements was taken as the final degree. Abnormal value was defined when the HT >2° (Hirvonen et al., 2011).

#### 2.4.3 OT

Fundus photographs were taken for both eyes using a nonmydriatic fundus camera. Before photography, patients were instructed to undergo 5 min of dark adaptation in a dark room to allow proper pupil dilation. Moreover, they were asked to keep their head in an upright position and look at a fixation target during photography. Patients were then asked to rest for 3 min with their eyes closed. After pupils returned to their normal size, the head position was kept unchanged, and photography for another eye was performed in the same way. Angle between a line connecting the optic disc center to fovea center and a horizontal line passing through the optic disc center was measured using image analysis software. Two photographs were captured by each eye, and the final degree is the average of the two photographs. Abnormal OT was considered if the difference in torsional angle between the two eyes was ≥8.8° (Choi et al., 2007).

#### 2.4.4 SD

Maddox rod was placed in the front of the patients’ right eye, and they were instructed to look at a light source at a distance of 33 cm with the both eyes. For the absence of SD, the dot and line that the patients saw were overlapped; for the presence of SD, the dot and line were separated from each other. After a prism was placed over the eyes, the patient complained that the separated dot and line are overlapped. The degree of prism was degree of SD (Green & Gold, 2021). Two experienced neurologists measured the SD separately, and the average of the two measurements was taken as the final degree. The time required for all 5 VGP evaluation is 20–30 min.

### 2.5 Ocular vestibular evoked myogenic potential (oVEMP)

oVEMP test was performed using the Interacoustics Eclipse system (Interacoustics, Middelfart, Denmark) in a conventional sound isolation room. Acoustic stimuli (intensity of 1000 dB nHL, short tone bursts of 500 and 1000 Hz) were delivered through insert earphones. The repetition rate was set to 5 times/sec with 200 stimuli repetitions/sweeps, and the recording window was 50 ms. The responses were bandpass filtered between 10 and 1000 Hz. The rise/fall time was 1 ms, and plateau time was 2 ms. During the test, patients were placed in a sitting position, and the local skin was cleaned. The recording electrodes were placed at about 1 cm below the midpoint of the bilateral infraorbital rim, the reference electrodes were placed 2–3 cm below the recording electrode, and the ground electrode was placed on the middle of forehead. Electrode impedance was maintained below 5 kΩ. Patients were instructed to maintain constant head position, elevate gaze to a fixed target about 1 m above the midline of the visual field at 30°. During recording, blinking and eye closure should be avoided to maintain stable extraocular muscle tone.

Normal bilateral amplitude asymmetry ratio was defined as ≤0.33. The normal oVEMP latency value defined by our laboratory was N13P18. No responses, amplitude asymmetry ratio falling outside of the normal range, or latency prolongation were considered abnormal results.

### 2.6 MRI protocol

A 3.0‐T MRI imaging scanner (Magnetom Avanto, Siemens, Germany) with an 8‐channel head coil was used to scan the entire temporal bone. MRI protocol was as follows: T1‐weighted imaging (T1WI), fast‐spin‐echo (FSE) T2‐weighted imaging (T2WI), and T2‐DRIVE‐HR plain in the transverse plane; enhanced T1WI in the transverse and coronal planes; 4‐h delayed gadolinium‐enhanced 3D FLAIR in the transverse plane. Gadopentetate dimeglumine (Gd‐DTPA, Magnevist, Bayer Healthcare) was used. All patients received a double dose of Gd‐DTPA (0.2 mmol/kg of body weight) via the antecubital vein injection with a high‐pressure syringe (Ulrich, Germany) at a rate of 2.0 mL/s, followed by intravenous push of 20 mL of saline. All patient data in this study were analyzed anonymously. The main scan parameters were as follows: T1WI: repetition time (TR), 687 ms; echo time (TE), 10 ms; thickness, 2.0 mm; slices, 16; time of acquisition, 2 min 4 s. FSE T2WI: TR, 3000 ms; TE, 90 ms; thickness, 2.0 mm; slices, 16; time of acquisition, 2 min 18 s. T2‐DRIVE‐HR: TR, 1500 ms; TE, 214 ms; thickness, 0.8 mm; slices, 64; time of acquisition, 3 min 3 s. 3D‐FLAIR: TR, 9000 ms; TE, 446 ms; inversion time, 1800 ms; thickness, 1.2 mm; slices, 24; time of acquisition, 9 min 2 s.

The endolymphatic hydrops standard is based on the Modified four‐stage vestibular EH grading system. Normal vestibule: The saccule and utricle are visible separately and take less than half of the surface of the vestibule. Vestibular hydrops grade I: The saccule, normally the smallest of the two vestibular sacs, has become equal or larger than the utricle but is not yet confluent with the utricle. Vestibular hydrops grade II: There is a confluence of the saccule and utricle with a peripheral rim enhancement of the perilymphatic space. Vestibular hydrops grade III: The perilymphatic enhancement is no longer visible. There is a full obliteration of the bony vestibule (Bernaerts et al., 2019). That is, when the saccule of the patient is equal or larger than the utricle, the patient is positive for endolymphatic hydrops.

### 2.7 Statistical analysis

Continuous variables were expressed as mean and standard deviation (mean  ±  SD), and comparisons of normally distributed and non‐normally distributed data were performed using independent sample *t*‐test and Mann–Whitney *U* test, respectively. Categorical variables are reported as percentages and were compared using the chi‐square test with Yates’ continuity correction or Fisher's exact test, as appropriate. All reported *p* values are two‐tailed, and *p* < .05 was considered statistically significant. All statistical analyses were performed with SPSS Statistics 25.0 (IBM SPSS Statistics, NY, USA).

## 3 RESULTS

### 3.1 Clinical baseline characteristics of all patients included in the study

This study enrolled 95 patients with UPVD, including 46 patients in the attack phase of UPVD and 49 patients in the remission phase. In the attack phase group, there were 21 males and 25 females, with an average age of 52.22 ± 13.59 years. In the remission phase group, there were 19 males and 30 females, with an average age of 46.84 ± 13.19 years. There was no statistical significance in sex, age, and the affected side of UPVD between the two groups (Table 1).

**TABLE 1:** The baseline characteristics of patients with unilateral peripheral vestibular dysfunction (UPVD) in the attack and remission phases

	Attack phase group (*n* = 46)	Remission phase group (*n* = 49)	*p* Value
Sex			.497
Male	21 (45.7%)	19 (38.8%)	
Female	25 (54.3%)	30 (61.2%)	
Age	52.22 ± 13.59	46.84 ± 13.19	.053
Affected side			.334
Left	18 (39.1%)	24 (49.0%)	
Right	28 (60.9%)	25 (51.0%)	

### 3.2 VGP evaluation results

#### 3.2.1 Results of qualitative analysis of VGP‐related indicators

**FIGURE 2:**
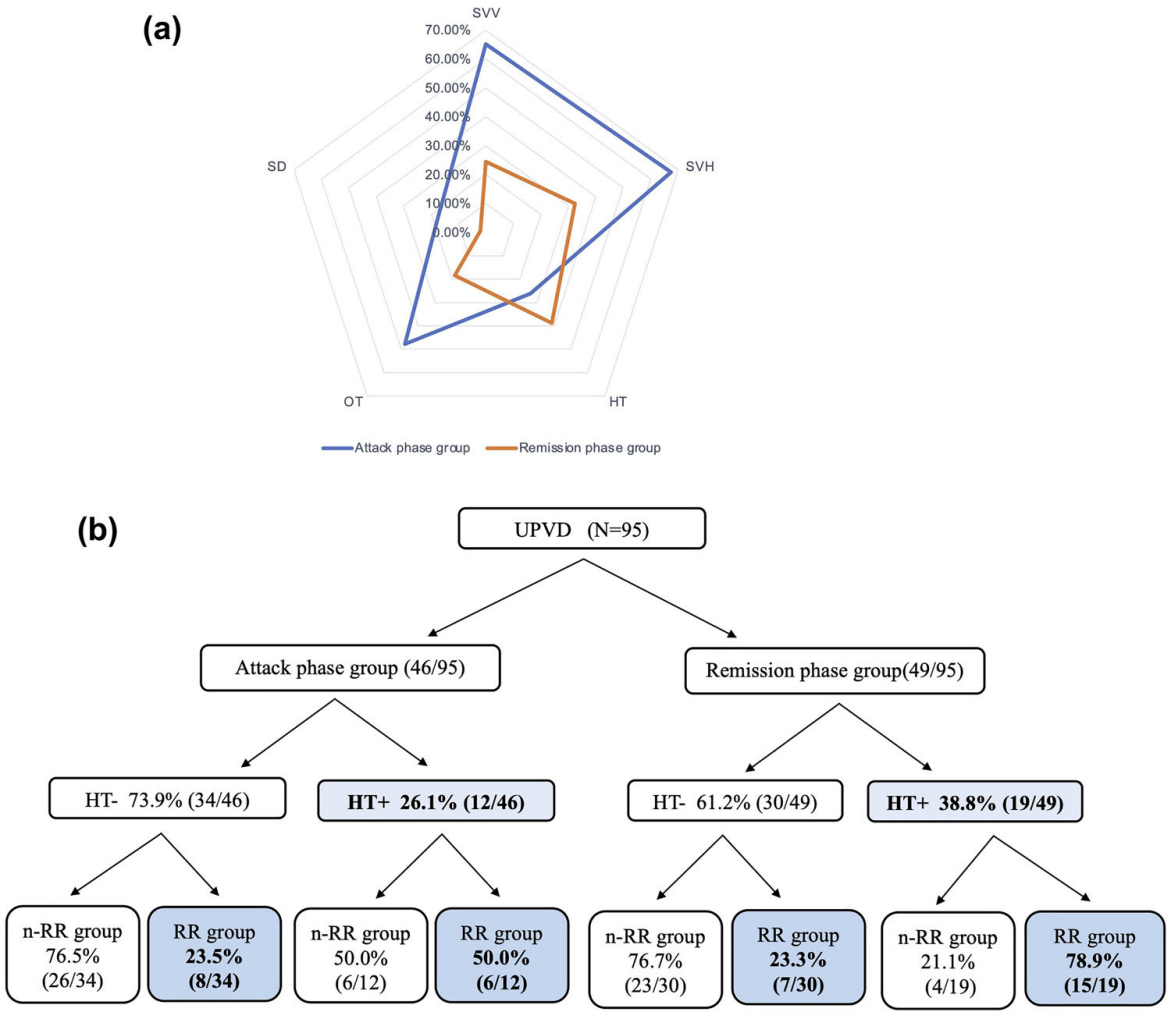
(a) Comparison of the positive rates of the five vestibular graviceptive pathway (VGP) evaluation indicators in patients with unilateral peripheral vestibular dysfunction (UPVD) between the attack and remission phase groups. (b) The association between the occurrence of ocular torsion (OT) and relapsing remitting phase of UPVD. HT, head tilt; nRR, non‐relapsing remitting; SD, skew deviation; SVH, subjective visual horizontal; SVV, subjective visual vertical.

The positive rates of SVV, SVH, OT, and SD were significantly higher in the attack phase group than in the remission group (SVV: 65.2% vs. 24.5%, *χ* ^2^ = 15.956, *p* < .001, Pearson chi‐square test; SVH: 67.4% vs. 32.4%, *χ* ^2^ = 11.454, *p =* .001, Pearson chi‐square test; OT: 47.8% vs. 18.4%, *χ* ^2^ = 9.366, *p =* .002, Pearson chi‐square test; SD: 17.4% vs. 2.0%, *p =* .012, Fisher's exact test). The positive rate of HT was higher in the remission phase group compared to the attack phase group, but the differences were not significant (26.1% vs. 38.8%, *χ* ^2^ = 1.738, *p =* .187, Pearson chi‐square test, Figure 2a). In order to analyze the reasons for this phenomenon, we further divided the patients into relapsing and non‐relapsing remitting groups according to their history of vertigo attacks and attack characteristics, and the results showed that the relapsing remitting phase of UPVD was significantly correlated with the presence of HT (*χ* ^2^ = 17.418, *p* < .001 Pearson chi‐square test; the attack phase group: *p =* .143 during attack, Fisher's exact test; the remission phase group: *χ* ^2^ = 14.543, *p* < .001, Pearson chi‐square test, Figure 2b).

#### 3.2.2 Results of quantitative analysis of VGP‐related indicators

**FIGURE 3:**
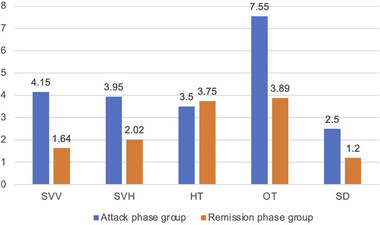
Comparison of the degrees of five vestibular graviceptive pathway (VGP) evaluation indicators in patients with unilateral peripheral vestibular dysfunction (UPVD) between the attack and remission phase groups. HT, head tilt; OT, ocular torsion; SD, skew deviation; SVH, subjective visual horizontal; SVV, subjective visual vertical.

The degrees of SVV, SVH, and OT were significantly greater in the attack phase group compared with the remission group (SVV: 4.15 ± 3.24 vs. 1.64 ± 1.71, *Z* = −4.277, *p* < .001; SVH: 3.95 ± 2.92 vs. 2.02 ± 1.45, *Z* = −3.878, *p* < .001; OT: 7.55 ± 6.30 vs. 3.89 ± 3.88, *Z* = −3.028, *p =* .002, Mann–Whitney test), whereas no statistically significant differences were found in the degrees of SVV precision, SVH precision, SD, and HT between the two groups (SVV: 1.05 ± 0.58 vs. 0.87 ± 0.53, *Z* = −1.764, *p =* .117; SVH: 0.94 ± 0.58 vs. 0.90 ± 0.48, *Z* = −0.321, *p =* .725; SD: 2.50 ± 1.60 vs. 1.00 ± 0.00, *Z* = 0.307, *p =* .444; HT: 3.83 ± 1.01 vs. 4.47 ± 1.35, *Z* = −1.553, *p =* .169, Mann–Whitney test, Figure 3).

#### 3.2.3 Correlations between VGP‐related indicators

Correlations among all the five indicators in VGP evaluation were found in patients with UPVD in the attack phase, whereas correlations between SVV and SVH were found in patients with UPVD in the remission phase (*r* = .505, *p* < .001, Table 2).

**TABLE 2:** Correlation between five vestibular graviceptive pathway (VGP) evaluation indicators in patients with unilateral peripheral vestibular dysfunction (UPVD)

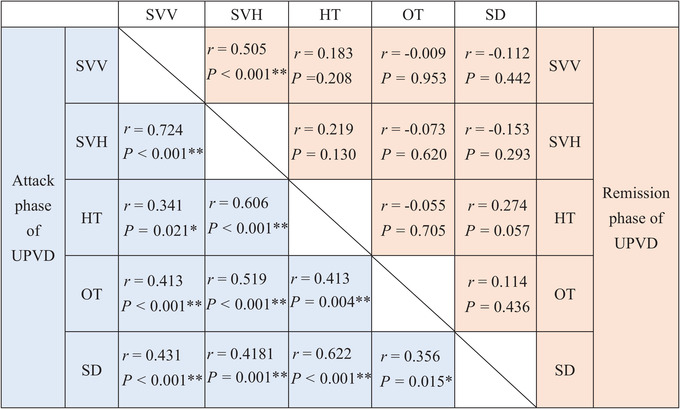

Abbreviations: *, *p* < 0.05; **, *p* < 0.01; HT, head tilt; OT, ocular torsion; SD, skew deviation; SVH, subjective visual horizontal; SVV, subjective visual vertical.

### 3.3 Correlations between VGP evaluation results and audiological, vestibular function test results

#### 3.3.1 Audiological test results

**FIGURE 4:**
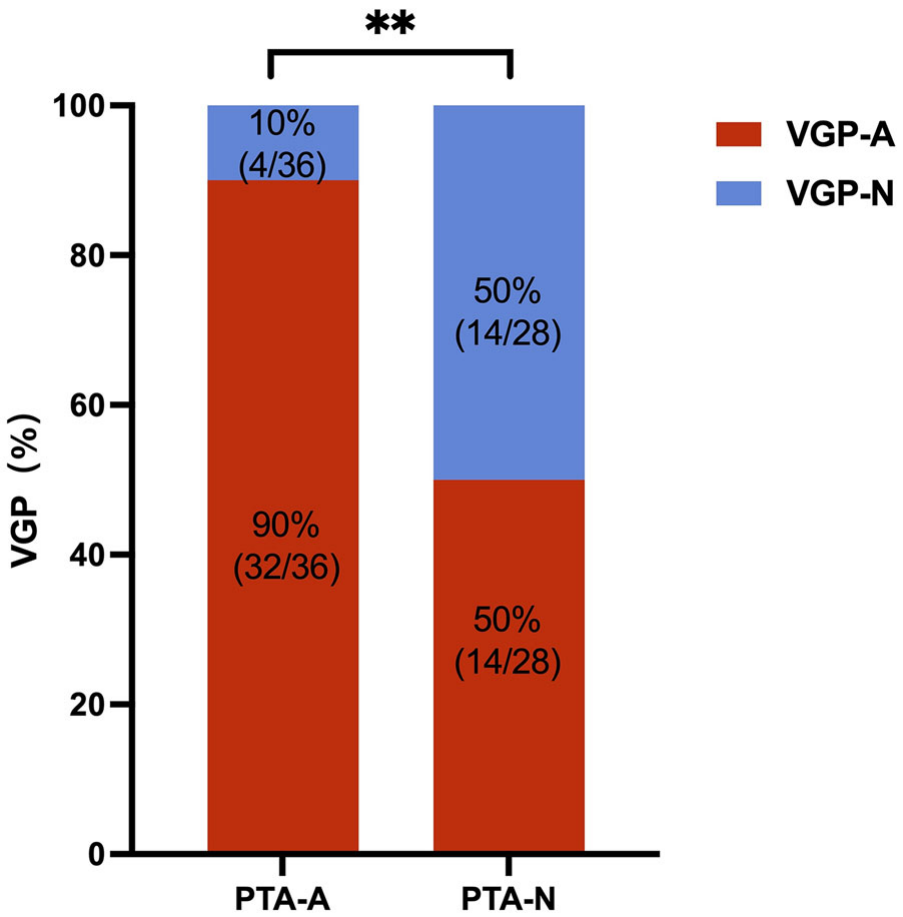
Correlations between vestibular graviceptive pathway (VGP) evaluation results and audiological test results. **, *p*<0.01; PTA‐A, pure tone audiometry‐abnormal; PTA‐N, pure tone audiometry‐normal; VGP‐A, vestibular graviceptive pathway evaluation‐abnormal; VGP‐N, vestibular graviceptive pathway evaluation‐normal.

Among 95 patients with UPVD included in the study, 64 patients underwent pure tone audiometry (PTA). PTA was calculated by averaging hearing threshold levels at 500, 1000, 2000, and 4000 Hz, and hearing loss was indicated if the PTA was >20 dB HL according to the international WHO standard classification (Human Rights Watch, 2017). Correlation analysis revealed a significant correlation between the sides with hearing loss and the sides with abnormal VGP evaluation results in both the two groups (*χ* ^2^ = 11.783, *p =* .001, Pearson chi‐square test; the attack phase group, *p =* .383, Fisher's exact test; the remission phase group, *χ* ^2^ = 10.898, *p =* .002, Pearson chi‐square test, Figure 4).

#### 3.3.2 Semicircular canal function test results

**FIGURE 5:**
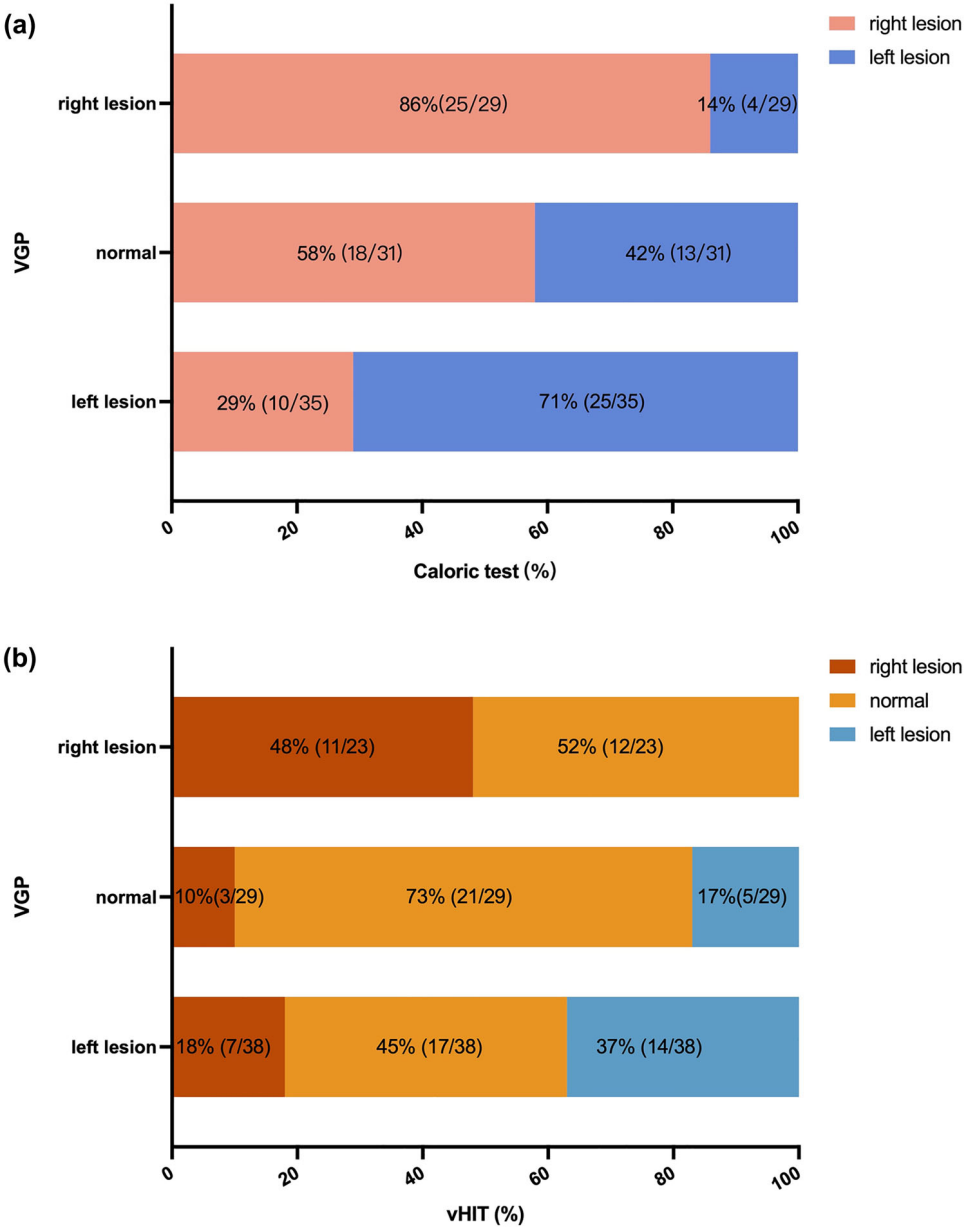
Correlations between vestibular graviceptive pathway (VGP) evaluation results and semicircular canal function test results: (a) There were significant correlations between the sides with abnormal caloric test results and the sides with abnormal VGP evaluation results; (b) there were significant correlations between the sides with abnormal VGP evaluation results and the sides with abnormal video head impulse test (vHIT) results.

Significant correlations between the sides with abnormal VGP evaluation results and the sides with abnormal caloric test results were found in both the two groups (*χ* ^2^ = 21.456, *p* < .001, Pearson chi‐square test; the attack phase group: *χ* ^2^ = 8.164, *p =* .015, Fisher's exact test; the remission phase group: *χ* ^2^ = 14.434, *p =* .001, Fisher's exact test, Figure 5a). There was no significant correlation between the degrees of all five VGP evaluation indicators and the CP value (SVV: *r* = .91, *p =* .379; SVH: *r* = .139, *p =* .178; OT: *r* = .088, *p =* .397; SD: *r* = .028, *p =* .787; HT: *r* = .065, *p =* .530).

Significant correlations were also found between the sides with abnormal VGP evaluation results and the sides with abnormal vHIT results in both the attack and remission phase groups (*χ* ^2^ = 20.151, *p* < .001, Fisher's exact test; the attack phase group: *χ* ^2^ = 11.586, *p =* .014, Fisher's exact test; the remission phase group: *χ* ^2^ = 5.152, *p =* .251, Fisher's exact test, Figure 5b).

#### 3.3.3 oVEMP test results

There was no significant correlation between the sides with abnormal VGP evaluation results and abnormal oVEMP results in either the attack phase group (*χ* ^2^ = 2.741, *p =* .626, Fisher's exact test) or the remission phase group (*χ* ^2^ = 2.837, *p =* .611, Fisher's exact test).

### 3.4 Clinical characteristics of UPVD patients with contralesional VGP (c‐VGP)

From the abovementioned correlation analysis between the VGP results and the caloric test results, we found that 14 (14/95, 14.7%) patients showed contralesional VGP (c‐VGP), that is, the sides with abnormal VGP evaluation results were opposite to the sides with abnormal caloric test results. In order to analyze the reasons for the presence of c‐VGP, we divided patients into a c‐VGP group and an ipsilesional VGP (i‐VGP) group and conducted further multidimensional evaluation.

#### 3.4.1 Clinical characteristics of UPVD patients with c‐VGP

Among the 14 UPVD patients with c‐VGP, 12 (85.7%) patients were in the attack phase, and 2 (14.3%) were in the remission phase. There was no statistical significance in the degrees and positive rates of VGP evaluation indicators, the degree of spontaneous nystagmus, and CP value in UPVD patients in both the attack and remission phases between the c‐ and i‐VGP groups (Table 3).

**TABLE 3:** Clinical characteristics of unilateral peripheral vestibular dysfunction (UPVD) patients in the contralesional VGP (c‐VGP) and ipsilesional VGP (i‐VGP) groups

		c‐VGP group (*n* = 14)	i‐VGP group (*n* = 81)	*p* Value
Attack phase group (*n* = 46)	**Clinical characteristics**			
	SN	5.08 ± 5.45	4.85 ± 4.81	.929
	**VGP**			
	SVV	4.80 ± 2.14 11 (91.7%)	3.93 ± 3.55 19 (55.9%)	.130 .059
	SVH	4.24 ± 2.12 10 (83.3%)	3.85 ± 3.17 21 (61.8%)	.374 .258
	HT	3.83 ± 0.62 4 (33.3%)	3.94 ± 1.07 8 (23.5%)	.559 .703
	SD	0 0 (0.0%)	2.50 ± 1.50 8 (23.5%)	– .090
	OT	6.33 ± 4.82 5 (41.7%)	7.99 ± 5.99 17 (50.0%)	.444 .742
	**Vestibular evaluation**			
	CP value	50.58 ± 24.04	56.41 ± 26.41	.540
Remission phase group (*n* = 49)	**VGP**			
	SVV	4.15 ± 4.74 1 (50.0%)	1.53 ± 1.51 11 (23.4%)	.310 .986
	SVH	3.35 ± 1.63 1 (50.0%)	1.97 ± 1.44 15 (31.9%)	.224 1.000
	HT	5.00 ± 1.41 2 (100%)	4.41 ± 1.33 17 (36.2%)	.071 .145
	SD	0 0 (0.0%)	1 1 (2.1%)	‐ 1.000
	OT	1.50 ± 2.12 0 (0.0%)	3.99 ± 3.92 9 (19.1%)	.383 1.000
	**Vestibular evaluation**			
	CP value	55.50 ± 19.09	43.0941 ± 15.07	.357

Abbreviations: CP, canal paresis; HT, head tilt; OT, ocular torsion; SD, skew deviation; SVH, subjective visual horizontal; SVV, subjective visual vertical; VGP, vestibular graviceptive pathway.

#### 3.4.2 Etiological analysis of c‐VGP

Multidimensional evaluation was performed on these 14 patients with c‐VGP to analyze the underlying etiologies. Among these 14 patients, 4 (4/14, 35.7%) UPVD patients had contralateral benign paroxysmal positional vertigo (BPPV), 8 (8/12, 66.7%) had bilateral hearing loss, 1 (1/13, 7.7%) had bilateral VOR gain reduction, 9 (9/12, 75.0%) had autoimmune diseases, 8 (8/14, 57.1%) had vascular risk factors, 3 (3/14, 21.4%) had lacunar infarction, and 3 (3/9, 33.3) patients showed affected endolymphatic hydrops on post‐contrast delayed 3D‐FLAIR MRI (Table 4).

**TABLE 4:** Multidimensional evaluation of etiologies of contralesional VGP (c‐VGP) in patients with unilateral peripheral vestibular dysfunction (UPVD)

								VGP					PTA				MRI		
No.	Gender	Age	Caloric test	SN	vHIT	oVEMP	cVEMP	SVV	SVH	HT	OT	SD	R	L	Vascular risk	Immunoassay	Cranial MRI	Internal auditory canal MRI	Other vestibular diseases
1	F	42	R (75%)	Lb	R lateral+ R posterior	N	N	L	L	L	L	N	20	18	A	A	N	R hydrolabyrinth	
2	F	33	R (76%)	Lb	R lateral+ L lateral	N	N	L	L	L	N	N	14	12	N	A	N	–	
3	M	60	R (27%)	Lb	N	N	N	L	N	N	N	N	27	26	N	N	N	R hydrolabyrinth	
4	M	59	R (32%)	Lb	N	N	N	L	L	N	N	N	29	34	N	A	N	N	
5	M	29	R (100%)	Lb+U	R lateral	N	N	L	L	N	N	N	–	–	A	A	N	–	
6	M	59	R (27%)	Lb	N	R	R	L	L	N	N	N	–	–	A	–	Lacunar infarction	–	
7	F	62	R (42%)	Lb+D	N	R	L	L	L	N	L	N	19^a^	21	A	A	N	–	LPC‐BPPV
8	F	75	R (29%)	Rb	N	R	N	L	L	N	N	N	30	29	N	N	Lacunar infarction	N	RPC‐BPPV
9	F	66	R (71%)	Rb+D	N	R+L	N	L	L	L	L	N	14	23	A	–	N	–	t‐BPPV
10	M	64	R (42%)	N	N	–	–	L	N	L	N	N	36	36	N	A	N	N	
11	F	60	L (36%)	Lb	N	L	N	R	R	R	R	N	21	21	A	A	N	N	RPC‐BPPV
12	M	66	L (44%)	D	N	N	N	N	R	N	N	N	21	20	A	A	Lacunar infarction	N	
13	M	63	L (48%)	Rb	–	N	N	R	N	R	R	N	19	68	A	A	N	N	
14	F	64	L (69%)	N	N	R+L	L	N	R	R	N	N	21	21	N	N	N	L hydrolabyrinth	RPC‐BPPV

Abbreviations: A, abnormal; BPPV, Benign paroxysmal positional vertigo; cVEMP, cervical vestibular evoked myogenic potential; D, downbeat nystagmus; F, female; HT, head tilt; L, left; Lb, left beating; LPC‐BPPV, left posterior semicircular canal‐BPPV; M, male; N, normal; OT, ocular torsion; oVEMP, ocular vestibular evoked myogenic potential; PTA, pure tone audiometry; R, right; Rb, right beating; RPC‐BPPV, right posterior semicircular canal‐BPPV; SD, skew deviation; SN, spontaneous nystagmus; SVH, subjective visual horizontal; SVV, subjective visual vertical; t‐BPPV, traumatic‐BPPV; U, upbeat nystagmus; VGP, vestibular graviceptive pathway; vHIT, video head impulse test.

^a^ The patient complained of obvious tinnitus symptoms in the left ear since the onset of the disease (there was no significant difference in hearing loss in both ears). Therefore, combined with clinical symptoms, even if the PTA hearing loss in the left ear did not reach the WHO standard classification of 20 dB, we also considered the patient to be qualified for bilateral hearing loss.

Comparison of age, vascular risk factors, and the incidence of immune abnormalities between the c‐ and i‐VGP groups showed that patients in the c‐VGP group were older than those in the i‐VGP group (54.14 ± 13.10 vs. 48.11 ± 13.30, *Z* = −2.316, *p =* .021, Mann–Whitney test). The prevalence of immune abnormalities was significantly higher in the c‐VGP group than in the i‐VGP group (75.0% vs. 21.1%, *p* < .001, Fisher's exact test), whereas there was no statistical difference in the prevalence of vascular risk factors between the two groups (57.1% vs. 34.6%, *χ* ^2^ = 2.585, *p =* .139, Pearson chi‐square test, Table 5). Logistic regression analysis was used to analyze whether age and immune abnormalities were independent risk factors for c‐VGP. The results showed that only immune abnormalities were identified statistically significant (OR = 9.710, 95%CI: 2.297–41.049, *p* = .002).

**TABLE 5:** Comparison of age, the prevalence of vascular risk factors and immune abnormalities in unilateral peripheral vestibular dysfunction (UPVD) patients between the contralesional VGP (c‐VGP) and ipsilesional VGP (i‐VGP) groups

		c‐VGP group (*n* = 14)	i‐VGP group (*n* = 81)	*p* Value
UPVD (*n* = 95)	Age	54.14 ± 13.10	48.11 ± 13.30	.021*
	Vascular risk factors	8/14 (57.1%)	28/81 (34.6%)	.139
	Immune abnormalities	9/12 (75.0%)	16/76 (21.1%)	.000*

Abbreviation: VGP, vestibular graviceptive pathway.

## 4 DISCUSSION

Previous studies (Brandt & Dieterich, 1995; Choi et al., 2007; Korda et al., 2022; Strupp, 2008; Zhao et al., 2017) have shown that for patients with UPVD in the attack phase, the positive rates of SVV, SVH, HT, OT, and SD were 50.6%−94%, 54.1%−91%, 4%−37%, 19%−82%, and 14%−24%, respectively, whereas for patients with UPVD in the remission phase, the positive rates of SVV, SVH, HT, OT, and SD were 0%−25%, 0%−22%, 0%−20%, 0%−20%, and 0%−5%, respectively, and these findings suggest that SVV, SVH, OT, and SD tend to return to normal range in patients with UPVD in the remission phase. In the present study, the results from VGP evaluation are quite similar to the abovementioned findings, and our results showed high positive rates of SVV and SVH. A previous study (Conrad et al., 2014) showed that in contrast to HT, OT, and SD, the body requires multiple peripheral, central vestibular pathways, and multisensory integration at high‐level nerve center to maintain the accuracy of SVV/SVH, such as vertical semicircular canal pathways, utricle‐mediated pathways, medial longitudinal fascicle pathway, brachium conjunctivum pathway, and ipsilateral vestibulothalamic tract pathway, as well as the integration of vestibular, proprioceptive, and visual information. We hypothesize that SVV/SVH is more sensitive and vulnerable than other VGP indicators, that is, damage at any site can lead to SVV/SVH abnormalities.

In this study, dynamic changes in SVV, SVH, OT, and SD were found in UPVD patients in both the attack and remission phases, whereas obvious change in HT was not noted. We speculate that this may be due to the following two reasons: (1) The body may be more sensitive to ocular position compensation and cortical perception than to vestibulo‐collic reflex (VCR) compensation. A study of patients with unilateral vestibular deafferentation after vestibular schwannoma resection conducted by Mantokoudis et al. (2014) found that compensation for SD was more quickly compared to dynamic VOR compensation. Aleisa et al. (2007) showed that compared with dynamic VOR, mice showed less compensation for VCR after unilateral labyrinthectomy. We speculate that after UPVD occurs, the body needs to restore eye position and perception in a timely manner to ensure clear vision and reduce vertigo/dizziness symptoms, thus participating in the compensation of the abovementioned indicators in a timely manner. (2) In contrast to the attack phase, the remission phase is not only a sign of damage to VGP but may also encompass the results of vestibular compensation. Physiologically, neuronal axons of the lateral vestibular nucleus project into the lateral vestibulospinal tract located ventrally in the spinal cord and synapse with interneurons in the ventral gray matter of the spinal cord. The medial vestibular nucleus sends fibers medially to the medial longitudinal fasciculus, which descends ventrally and bilaterally in the spinal cord to the mid‐thoracic level as the medial vestibulospinal tract. The interneurons are facilitatory to the alpha and gamma motor neurons of the ipsilateral extensor muscles of the limb and inhibitory to flexion of the limb. Additionally, interneurons project to the contralateral gray matter of the spinal cord and inhibit the contralateral extensor muscles of the limb, so vestibular system activation on one side can lead to increased ipsilateral extensor tone and decreased contralateral extensor tone. When peripheral vestibular function is impaired on one side in patients, the contralateral peripheral vestibular nucleus becomes relatively excited, causing the patients’ head to tilt to the affected side (Kent et al., 2010). In the present study, we found that the relapsing remitting phases of UPVD were associated with HT. Asama et al. (2012) showed that vestibular compensation for VCR occurred in patients with chronic dizziness, further increasing proprioceptive afference from the neck muscles to the vestibular nucleus, they believe that the asymmetry of the neck muscle tension could reflect the bilateral asymmetry of vestibular function, and the increase in the asymmetry of the neck muscle tension is intended to compensate for postural stability, that is, vestibular compensation can cause HT in patients after the peripheral vestibular function is impaired on one side. Further dynamic studies with a large sample size are needed to explore the relationship between HT and disease duration.

In terms of correlation, 5 VGP evaluation indicators showed a pairwise correlation in the attack phase group, whereas in the remission phase group, only SVV and SVH were correlated, which reflected the dynamic changes of the VGP evaluation indexes from the side, that is, the VGP evaluation indicators that respectively reflect vestibule‐cervical, vestibule‐ocular, and cortical perception have different compensation speeds in different periods. In previous studies, it is generally believed that SD and HT recover quickly and usually improve within 4–8 weeks (Hirvonen et al., 2011; Strupp, 2008). There is controversy over the recovery speed of SVV and OT. Studies have shown that both SVV and OT can be improved between 1 and 6 months (Choi et al., 2007). Some studies suggest that OT has a longer recovery time than SVV and can last for a year (Faralli et al., 2021). In addition, some studies also believe that SVV can exist in chronic UPVD in a small but persistent manner for many years (Müller et al., 2016). As the conduction pathways of SVV and SVH both start from the vertical semicircular canals and utricle, and final project to the thalamus and PIVC participate in the formation of space perception, their essence is the same. Moreover, the correlation between the two in the VGP tests has been continuously verified in previous studies, and it is generally believed by scholars that any of the tests can be used clinically. Therefore, SVV and SVH have a reasonable correlation in different periods of UPVD.

The results of this study also revealed that the sides with abnormal VGP evaluation results were closely correlated with the sides with reduced vestibular function or hearing loss, suggesting that VGP evaluation might be useful to determine the affected side in patients with UPVD, and the affected side determined by VGP evaluation can be corroborated with the findings from audiological and semicircular canal function tests. However, oVEMP appears not to determine the affected side of UPVD. Our results revealed no correlation between the sides with abnormal oVEMP test and the sides with abnormal VGP evaluation results either in the attack group or in the remission group. Controversy still exists over whether oVEMP test results are consistent with VGP evaluation results. A study of 138 patients with acute dizziness/vertigo conducted by Hösli and Straumann (2021) showed that there was no correlation between oVEMP and OT, SVV results. Similarly, Nagai et al. (2014) analyzed the correlation between oVEMP and SVV results in 109 patients with acute unilateral inner ear diseases (VN, sudden sensorineural hearing loss, and Menière's disease [MD]) and found no correlation between the oVEMP and SVV. Lin and Young (2011) investigated the correlation between oVEMP and SVH tests in 20 healthy subjects and 20 patients with MD and found that oVEMP test results were significantly correlated with SVH results in both healthy subjects and MD patients. A study of 43 patients with acute VN conducted by Taylor et al. (2016) documented a significant correlation between oVEMP and SVH. The inconsistency between oVEMP test results and VGP evaluation results may be due to the following two reasons: (1) oVEMP test results and VGP evaluation may have assessed different aspects of utricular function. It is known that there are two types of receptors in the utricle that are sensitive to static and dynamic stimuli, respectively. The selectivity for the two receptors may be different when the utricle is damaged, thus leading to inconsistency between oVEMP and VGP; (2) both the two tests are confounded by other factors, that is, neither the VEMP test nor VGP evaluation assess the utricular function purely. oVEMP contains afferents from the labyrinth, and OT contains afferent information from the vertical semicircular canal.

Interestingly, in this study, we found the presence of c‐VGP in some of the UPVD patients. It is hypothesized that the presence of c‐VGP in UPVD patients may be associated with bilateral labyrinthine lesions or endolymphatic hydrops on the affected side. In this study, we performed a multidimensional assessment based on medical history‐physical examination‐related vestibular function tests‐laboratory examinations in UPVD patients who showed c‐VGP in order to explore the possible etiologies. The results showed that among UPVD patients with c‐VGP, 35.7% of these patients had contralateral BPPV, 33.3% showed affected endolymphatic hydrops on post‐contrast delayed 3D‐FLAIR images, and evidence of bilateral labyrinthine lesions was found in the remaining 57.1% of the patients (eight cases (8/12, 66.7%) with bilateral hearing loss, one case (1/13, 7.7%) with decreased bilateral VOR gain, nine (9/12, 75.0%) had autoimmune diseases, eight (8/14, 57.1%) had vascular risk factors). The occurrence of c‐VGP does not seem to be uncommon in UPVD patients. Faralli et al. (2014) evaluated SVV in seven patients with first attack of MD and found that the SVV was directed toward the ipsilateral side in three patients, which was directed toward the healthy side in the remaining four patients. Kumagami et al. (2009) assessed the SVH in 14 patients with MD in the acute attack phase and found SVH tilt toward the contralateral side in one patient. A retrospectively study of 52 patients with a contraversive SVH conducted by Nham et al. (2020) showed that 67.3% of these patients had MD, 7.7% had delayed endolymphatic hydrops, and 25% had unclear etiologies, so it is speculated that c‐VGP suggests endolymphatic hydrops. A study of 51 patients with VN conducted by Kim and Hong et al. (2008) also observed the occurrence of c‐VGP in 2 patients. The reason for the presence of c‐VGP in patients with endolymphatic hydrops may be related to the increased activity of the utricle caused by hydrops stimulation on the affected side. In addition, we further analyzed the possible causes of bilateral labyrinthine lesions and found that autoimmune abnormalities associated with Hashimoto's thyroiditis were one of the possible causes of c‐VGP. Hashimoto's thyroiditis is characterized by the presence of thyroglobulin antibodies in the serum. Thyroid peroxidase antibody (TPOAb) and thyroglobulin antibody were found to be positive in 90% of patients with Hashimoto's thyroiditis (Gunes et al., 2017). Circulating autoimmune antibodies can cause damage to the labyrinth and connective tissues in various organs throughout the body. Chiarella et al. (2014) showed that up to 44.7% of patients with Hashimoto's thyroiditis showed unilateral CP during caloric test, and 55.2% of the patients showed abnormal VEMP. Gawron et al. (2004) showed that in patients with Hashimoto's thyroiditis, auditory nerve and brainstem neural conduction in the brain auditory evoked potentials were both affected, and TPOAb titers were positively correlated with the extent of central hearing disorder. It can be seen that autoimmune abnormalities associated with Hashimoto's thyroiditis might be one of the possible causes of c‐VGP in UPVD patients.

The study has some limitations. This was a single‐center study with small sample size, so there is a possibility of selection bias. In addition, dynamic follow‐up study on UPVD patients was not performed in the study. Further studies with larger sample size and dynamic follow‐up are needed to explore the clinical application value of VGP evaluation in the diagnosis of UPVD.

## 5 CONCLUSIONS

Our findings suggest that (1) alterations in SVV, SVH, OT, and SD were noted in UPVD patients in the acute attack and remission phases, which are presumed to be related to dynamic vestibular compensation; (2) there were significant correlations between VGP evaluation results and semicircular canal function test, audiological test results, indicating that VGP evaluation might be also useful to determine the affected side in UPVD patients; (3) the presence of c‐VGP in UPVD patients may be related to bilateral labyrinthine lesions or endolymphatic hydrops on the affected side, and the involvement of autoimmune mechanisms also deserves attention.

## AUTHOR CONTRIBUTIONS

Xiao‐hong Ba, Xu Yang contributed to the conception and design of the study. Tong‐tong Zhao collected the data, analyzed the results, and drafted the manuscript. Meng‐lu Zhang, Yu‐fei Feng, Qian‐qian Wang, and Ning Song collected the clinical data and analyzed the results. All authors contributed to the article and approved the submitted version.

## CONFLICT OF INTEREST STATEMENT

The authors declare no competing interests.

### PEER REVIEW

The peer review history for this article is available at https://publons.com/publon/10.1002/brb3.3055.

## Data Availability

The data used in this study are available from the corresponding author, upon reasonable request.
